# Toll-like receptor 9 interaction with CpG ODN – An *in silico* analysis approach

**DOI:** 10.1186/1742-4682-10-18

**Published:** 2013-03-14

**Authors:** Wei Zhou, Yan Li, Xichun Pan, Yuan Gao, Beiping Li, Zhengliang Qiu, Long Liang, Hong Zhou, Junjie Yue

**Affiliations:** 1 Beijing Institute of Biotechnology, Beijing 100071, China; 2 Hospital No.307 of PLA, The Academy of Military Medical Sciences, Beijing 100071, China; 3 College of Pharmaceutical, Third Military Medical University, Chongqing 400038, China; 4 Laboratory Animal Center of the Academy of Military Medical Sciences, Beijing 100071, China

## Abstract

**Background:**

Toll-like receptor 9 (TLR9) recognises unmethylated CpG DNA and activates a signalling cascade, leading to the production of inflammatory cytokines such as TNF-α, IL-1, IL-6 and IL-12 via the adaptor protein MyD88. However, the specific sequence and structural requirements of the CpG DNA for the recognition of and binding to TLR9 are unknown. Moreover, the 3D structures of TLR9 and the TLR9-ODN complex have not been determined. In this study, we propose a reliable model of the interaction of the TLR9 ECD with CpG ODN using bioinformatics tools.

**Results:**

The three-dimensional structures of two TLR9 ECD-CpG ODN complexes were constructed using a homology modelling and docking strategy. Based on the models of these complexes, the TLR9 ECD-CpG ODN interaction patterns were calculated. The results showed that the interface between the human TLR9 and the CpG ODN molecule is geometrically complementary. The computed molecular interactions indicated that LRR11 is the main region of TLR9 that binds to CpG ODN and that five positively charged residues within LRR11 are involved in the binding of the TLR9 ECD to the CpG ODN. Observations in the close-up view of these interactions indicated that these five positively charged residues contribute differently to the binding region within the TLR9 ECD-CpG ODN complex. 337Arg and 338Lys reside in the binding sites of ODN, forming hydrogen bonds and direct contacts with the CpG ODN, whereas 347Lys, 348Arg, and 353His do not directly contact the CpG ODN. These results are in agreement with previously reported experimental data.

**Conclusion:**

In this study, we present two structural models for the human and mouse TLR9 ECD in a complex with CpG ODN. Some features predicted by this model are consistent with previously reported experimental data. This complex model may lead to a better understanding of the function of TLR9 and its interaction with CpG ODN and will improve our understanding of TLR9-ligand interaction in general.

## Background

Toll-Like Receptors (TLRs) recognise pathogen-associated molecular patterns (PAMPs) and are thought to be the key sensors of invading microbes in the innate immune system. Thirteen TLR members (TLR1-13) have been identified that are expressed on the cell surface (TLR 1, 2, 4, 5, and 6) or within the endosomal compartment (TLR 3, 7, 8, and 9). The surface-expressed TLRs primarily recognise structural components of pathogens, while the endosomal TLRs are dedicated to recognising nucleic acids [1].

The TLRs are type I integral membrane glycoproteins that consist of a pathogen-binding ectodomain (ECD) and a cytoplasmic signalling domain, joined by a single transmembrane helix [2]. Pathogen-binding ectodomains of mammalian TLRs comprise 19–25 extracellular leucine-rich repeats (LRRs) and a cytoplasmic toll/interleukin (IL)-1R (TIR) domain [3]. LRRs containing 24–29 amino acids are responsible for ligand recognition and binding, and the TIR domain is responsible for downstream signalling.

TLR9 is a receptor for sensing bacterial DNA/CpG-containing oligodeoxynucleotides (CpG ODN) within the endosomal compartment [4]. Internalised CpG DNA within the endosome initiates TLR9-mediated signalling via the sequential recruitment of MyD88, IRAK and TRAF6, which in turn activate important downstream transcription factors such as NF-κB and AP-1. These transcription factors induce the expression of inflammatory cytokines such as TNF-α, IL-6, IL-1β and IL-12 [5]. There are 25 LRRs in the ECD of human TLR9 (hTLR9); however, it is unknown which LRRs recognise and bind CpG DNA and which specific amino acids within the LRR are the most important for mediating recognition and binding.

Many studies have explored the mechanism of CpG DNA binding to its receptor. In 2003, LRRs 2, 5, and 8, bearing long insertions at position 10 following the consensus Asn residue, were thought to be the recognition sites of TLR9 [6]. LRR8 contains a 6-residue insert, similar to a motif identified in a protein that directly binds to unmethylated CpG dinucleotide sequences; thus, LRR8 was thought to be a site of CpG DNA recognition by TLR9 [7]. It was recently proposed that cleavage of the ECD occurs in mouse TLR9 (mTLR9) and that the fragment starting from LRR15 mediates ligand recognition [8,9].

In 2009, Peter et al. found that LRRs 2, 5, and 8 contribute to CpG DNA-induced activation of TLR9 and that deletion of the inserted sequences at position 10 leads to a loss of receptor binding ability. They also identified a positively charged region of the N-terminus that was essential for CpG DNA-induced TLR9 activation [10]. Another finding indicated that two variants of hTLR9, Pro99Leu within LRR2 and Met400Ile within LRR13, are associated with altered receptor function with regard to NF-κB activation and cytokine induction [11], suggesting that the N-terminal fragment preceding LRR14 also binds to CpG ODN.

Although the above reports provide useful information about the recognition and binding of CpG ODN to TLR9, detailed information about the precise sequence and structural requirements of the TLR9 interaction with CpG ODN is lacking. It remains unclear which LRR on TLR9 is the binding site(s) for the CpG ODN are which residues within the LRR directly contribute to the binding, primarily because of the lack of structural information on either TLR9 or the TLR9-ODN complex.

The aim of this study is to define a structural model for the TLR9-CpG ODN complex and to describe the interaction between TLR9 and CpG ODN using computational methods. Therefore, we can identify and characterise the LRR region within hTLR9 that binds to CpG ODN with the higher affinity and further determine which specific residues are critical for ligand binding.

## Results

### Residues within LRR11 in hTLR9 provide the greatest number of interactions with CpG ODN

The TLR9-ODN complex was modelled in three steps. First, we predicted the structure of the TLR9 ECD using a homology modelling approach. Second, we built the ODN in substrate mode. Third, we docked the CpG ODN substrate to the protein model.

The computed hTLR9 ECD model revealed a horseshoe-shaped assembly that adopted an irregular solenoid structure with some disordered regions. The compatibility of the hTLR9 ECD structure with the amino acid sequence was assessed by the Profiles-3D program in Discovery studio 3.0. The Verify Score of this predicted hTLR9 ECD model was 275.1, which is higher than the Verify Expected Low Score (164.1). This result indicates that the model is of acceptable quality.

**Figure 1:**
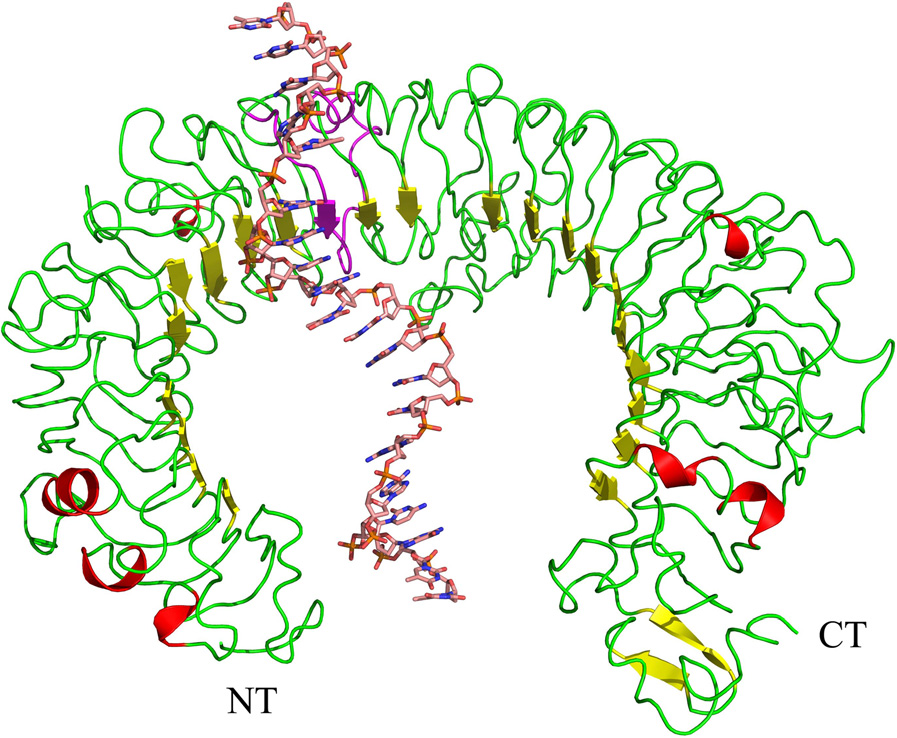
**The structure of the hTLR9-ODN complex obtained by the Protein-DNA docking method.** The structure of hTLR9 ECD is shown in carton; helical residues and beta sheet residues are coloured yellow, and the loop and unassigned residues are coloured green. LRR11 is coloured magenta. CpG ODN is shown in rainbow sticks.

The default protein-DNA docking protocol implemented in Hex 6.12 was used for the docking runs. The final complex model was characterised in terms of the interaction features to improve our understanding of the mechanism of hTLR9-CpG ODN recognition. The interaction pattern showed that the hTLR9 ECD-CpG ODN interaction sites were located close to the central region of the hTLR9 ECD (Figure 1). The interface between the receptor and the CpG ODN molecule was geometrically complementary. The interface area of the modelled complex was approximately 1790 Å^2^, with a buried surface area of 1720±760 Å^2^, suggesting that the complex had the “standard-size” interface of a protein and single-stranded DNA complex [12].

Because of the considerable size of CpG ODN, the ligand-binding region of hTLR9 contained many residues. The residues involved in the protein-nucleic acid interface of the hTLR9 ECD-CpG ODN complex were determined on the basis of the buried surface area. There were about 25 residues that lost their accessible surface in the hTLR9-CpG ODN complex. These residues constituted a large contact area to bind the CpG ODN molecule. The interface area contributed by the protein was 840 Å^2^, which was 46.9% of the total interface area. Among the 25 residues of hTLR9, residues 224Tyr, 248Val, 290Val, 312Val, 314Asp, 337Arg, 338Lys, 340Asn, 364Val, 365Ala, 367Lys, 392Met, and 397Arg were important for ligand recognition, and they established more direct and closer contact with the CpG ODN molecule.

In their review, Rohs et al. proposed a new theory regarding the origin of specific recognition between proteins and DNA [13]. They hypothesised that hydrogen bonds and hydrophobic contacts are principally responsible for the recognition specificity. Hydrogen-bond interactions are likely the main force dictating DNA sequence reading by proteins [14]. The hydrophobic bases are more exposed in single-stranded DNA than in dsDNA, leading to more hydrophobic interactions [15,16]. We examined how these interactions affect the binding of protein and DNA by analysing the distribution of these interactions throughout the hTLR9 ECD-CpG ODN complex.

There are 7 intermolecular hydrogen bonds and 29 hydrophobic interactions in the hTLR9 ECD-CpG ODN complex. The hydrogen bonds and hydrophobic interactions in this complex model are listed in Table 1 and Table 2, respectively. It has been shown that 90% of the protein-nucleic acid hydrogen bonds have the donor group on the protein and the acceptor group on the DNA [12]. Similarly, five of the seven hydrogen bonds in the hTLR9 ECD-CpG ODN complex have the donor group on the protein, and the other two have the donor group on the ODN.

**Table 1:** Hydrophobic interactions observed in the complex between human TLR9 and the ODN

**Amino acid**	**Atoms**	**Nucleotide**	**Atoms**
TYR224	CZ	C8	C3^′^
VAL248	CG1	C8	C5^′^
VAL290	CG2	G9	C5^′^
VAL312	CB	G6	C2
VAL312	CG1	C8	C5^′^
VAL312	CG2	A7	C2
ASP314	CB	G9	C5^′^
ARG337	CD,CZ	A4	C2,C5,C6
ARG337	CZ	T5	C2
ARG337	CB,CZ	G6	C2,C4,C5,C6
LYS338	CD	A7	C2
LYS338	CD,CE	C8	C2
LYS338	CE	G9	C2
ASN340	CG	T10	C5^′^
VAL364	CG1	C3	C5
ALA365	CB	A4	C6
LYS367	CE	T5	C4,C5
MET392	CE	C3	C2^′^,C3^′^
ARG397	CZ	T11	C5^′^

**Table 2:** Hydrogen bond interactions observed in the complex between human TLR9 and the ODN

**Donor**	**Acceptor**	**Distance**	**Angle**
A:TYR224:HH	S:DG9:OP1	2.18	121.3
A:ARG337:HH22	S:DT5:O2	2.48	86.1
A:LYS338:HZ2	S:DC8:O2	2.06	126.2
A:LYS338:HZ2	S:DG9:N3	2.43	107.2
A:LYS367:HZ1	S:DT5:O4	1.67	129.8
S:DC3:H42	A:GLN335:OE1	2.11	130.1
S:DG6:H22	A:ARG311:O	2.21	165.8

Some residues within LRR11, such as 335Asn, 337Arg, 338Lys, and 340Asn, formed more strong interactions with ODN 1826. Thus, LRR11 may be the main region of hTLR9 that binds to CpG ODN. This result is consistent with the experimental data in a prior report. We previously demonstrated that LRR11 peptide could bind to CpG ODN with high affinity, significantly decreasing CpG ODN internalisation and subsequent CpG ODN/TLR9 signalling. The hTLR9 mutant with a deletion in LRR11 conferred decreased responses to CpG ODN in HEK293T cells. These results suggest that LRR11 strongly binds to CpG ODN and that LRR11 may be the main region of hTLR9 that binds to CpG ODN [17].

### Five positively charged residues of LRR11 contribute differently to the binding of hTLR9 to CpG ODN

Analysis indicates that the interfaces where protein interacts with DNA are highly enriched in positive charges and almost devoid of negative charges [14]. The positively charged lysine and arginine account for 41% of the average interface area, while the negatively charged aspartic acid and glutamic acid only account for 4% of the interface area [12]. In the predicted hTLR9 ECD-CpG ODN complex model, LRR11 was the main region of hTLR9 binding to CpG ODN. Sequence analysis revealed there are five positively charged residues within LRR11 of hTLR9.

In our previous report [17], mutational studies indicated that a mutation at any one of the five residues drastically decreased the affinity of LRR11 for ODN, and among the five single mutations, R337S and K338N led to the most severe losses of LRR11-ODN binding. These results demonstrated that all five positively charged residues in LRR11 were essential for the high affinity binding of LRR11 with CpG ODN but that 337Arg and 338 Lys contributed more than the other residues to the binding affinity.

**Figure 2:**
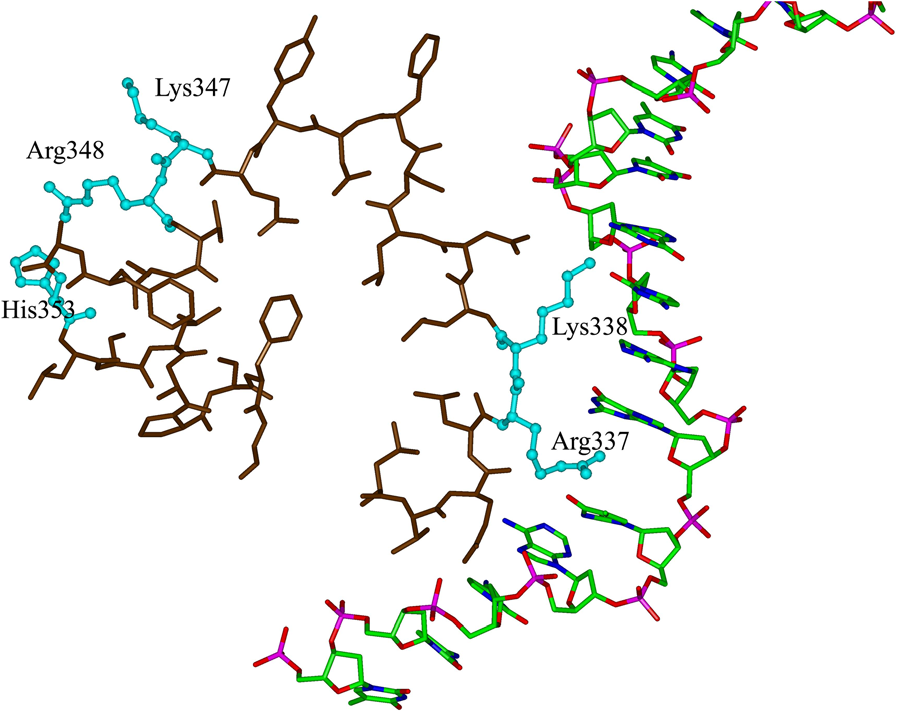
**Close-up views of the interactions between hTLR9 and the ODN.** TLR9 is depicted in violet; Arg337, Lys338, Lys347, Arg348 and His353 are highlighted in cyan and are shown as sticks and balls. Among the five positively charged residues, only Arg337 and Lys338 were in close contact with the CpG ODN molecule.

The docked structure provided insight into the role of the five positively charged residues in the interaction with the ODN. The computed hTLR9 ECD-CpG ODN complex showed that Arg337 and Lys338 were located on the concave face of the horseshoe and that they were the binding sites of the ODN. In the closed view of the binding region within the complex, the hTLR9 ECD anchored the ODN strand with Arg337 and Lys338; the other three positively charged residues Lys347, Arg348 and His353 were not directly involved in binding to the ODN (Figure 2) but were located in the insertion loop that connected the β-face with the convex surface. The molecular interaction details showed that Arg337 and Lys338 contributed the main hydrogen bonds in the hTLR9 ECD-CpG ODN complex and formed direct hydrophobic contacts with the ODN. Although the three other positively charged residues, Lys347, Arg348 and His353, did not directly interact with the ODN molecule, mutations at these positions caused rearrangement of the loop structure of LRR11. The effect of this rearrangement would propagate to the nearby LRRs, distorting the shape of the TLR9 interface surface.

As seen in all protein-DNA complexes, the interface is geometrically complementary between the protein and DNA molecules, ensuring an optimal intermolecular contact [14]. The complementary interface between the hTLR9 ECD and the ODN molecule is essential for their interaction. The shape of the TLR9 interface surface serves as a discriminating factor for ODN recognition; any alteration of TLR9 surface properties would influence ligand binding.

A similar phenomenon was reported by others for the D299G polymorphism of TLR4. The D299G SNP is located in the loop region of LRR10, on the convex face of TLR4. In a recent report, the substitution of more flexible and neutral residues, such as Asp299 to Gly299, induced a structural change in the loop region of LRR10-12, which caused the modulation of TLR4 surface properties and affected ligand binding [18].

### There are more hydrophobic interactions between mouse TLR9 and the CpG ODN than between human TLR9 and the CpG ODN

Species-specific ligand binding exists for many TLRs. For example, human and murine TLR2 can be activated by different lipoproteins [19]. Species-specific differences have been observed in the recognition of LPS between mammalian species [20-23]. Similarly, TLR9s in different vertebrate species have distinct CpG ODN specificities; human and mouse TLR9s display distinct response profiles to CpG-ODN (24, 25). CpG-ODN containing a GTCGTT motif preferentially activate hTLR9, whereas CpG-ODN with a GACGTT motif more strongly activate mTLR9. Because it contains a GACGTT motif, CpG-1826 more potently activated mTLR9 than it activated hTLR9 [24,25].

The factors underlying the receptor’s species specificity remain unknown. The cause of the activation discrepancy between human and mouse TLR9 for CpG ODN −1826 is still unclear. To this end, comparative homology modelling and docking studies were employed to construct a mTLR9 ECD-CpG ODN complex model. We compared the structure of the mTLR9 ECD-CpG ODN complex with that of hTLR9 ECD-CpG ODN complex and analysed the changes in the intermolecular interactions of the two complexes.

**Figure 3:**
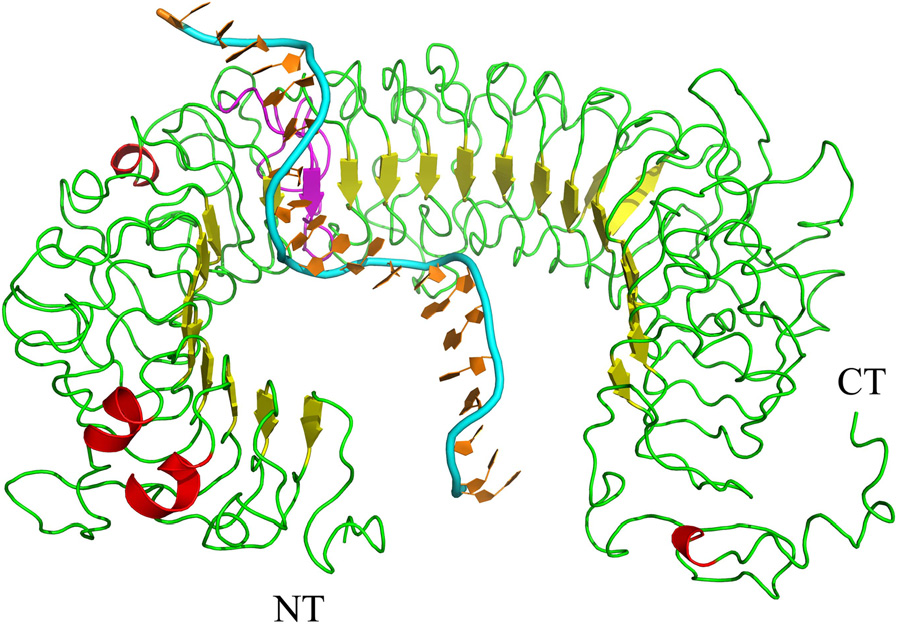
**Proposed mTLR9 ECD-CpG ODN complex.** Both the mTLR9 ECD and the CpG ODN are shown in carton. The N-terminus (NT) and C-terminus (CT) of the mTLR9 ECD are indicated, with LRR11 highlighted in magenta.

The pattern of mTLR9 interaction with the CpG ODN exhibited both similarities to and differences with that of hTLR9 (Figure 3). The hydrogen bonds and hydrophobic interactions in the mTLR9 ECD-CpG ODN complex are listed in Table 3 and Table 4, respectively.

**Table 3:** Hydrophobic interactions observed in the complex between mouse TLR9 and the ODN

**Amino acid**	**Atoms**	**Nucleotide**	**Atoms**
Tyr224	CE1,CZ	C13	C3^′^
Val248	CG1	T14	C7
Glu287	CB,CG,CD	T14	C4,C5,C7
Ser311	CB	T14	C4
Ser311	CB	G15	C6
Val312	CG2	C12	C5
Val312	CB,CG1,CG2	C13	C5, C6
Val312	CB,CG1	T14	C4
Thr334	CB,CG2	G18	C4^′^,C1^′^
Thr334	CB,CG2	T19	C5^′^,C4^′^
Arg335	CA,CB,CG,CD	A16	C2,C6
Arg335	CB,CG,CD,CZ	C17	C2,C4
Arg335	CD,CZ	G18	C2
Arg337	CB,CG,CD,CZ	G15	C2,C6
Arg337	CZ	C17	C4^′^,C5^′^
Lys338	CB,CG	C13	C4
Lys338	CG	T14	C2
Phe343	CE1	T10	C5^′^
Val364	CG2	G18	C5^′^
His397	CG,CD2,CE1	T10	C7
His397	CE1	G9	C2^′^,C3^′^
Gln399	CD	G9	C5^′^

**Table 4:** Hydrogen bond interactions observed in the complex between mouse TLR9 and the ODN

**Donor**	**Acceptor**	**Distance**	**Angle**
A:TYR224:HH	S:DC13:OP1	2.41	133.1
A:TYR224:HH	S:DC13:O5*	1.84	149.3
A:SER311:HG	S:DG15:O6	2.11	111.5
A:LYS338:HZ2	S:DT14:O2	2.28	124.7
A:ASN370:HD22	S:DT10:OP1	2.15	130.5
A:LYS392:HZ1	S:C17:O3*	2.48	152.6
S:DG15:H22	A:ARG337:NE	2.44	105.8

The predicted mTLR9 ECD-CpG ODN complex had an interface area of about 1945 Å^2^, which is greater than that of the hTLR9 ECD-CpG ODN complex. The interface area measures the area of protein and nucleic acid surface that is buried in contacts between the two macromolecules. The extent of the contact between two macromolecules is indicated by the size of interface area. In the mTLR9 ECD-CpG ODN model, 31 amino acid residues of mTLR9 and 13 nucleotide residues of CpG ODN 1826 were included in the interface. Among the 31 residues of mTLR9, 6 residues formed hydrogen bonds to the CpG ODN, while 13 residues established hydrophobic contacts with the CpG ODN. More accessible interface area increased the probability of an interaction between TLR9 and CpG ODN.

Seven intermolecular hydrogen bonds directly linking the TLR9 protein to the CpG ODN were observed for both complexes, while more hydrophobic contacts were observed in the mTLR9 ECD-CpG ODN complex (Table 5). Since the difference in hydrophobic interactions was great, the increased number of intermolecular hydrophobic contacts might promote binding stability. These results indicate that mTLR9 has a tighter binding with CpG ODN 1826 compared to hTLR9; hence, we hypothesised that the disparity in the number of hydrophobic interactions may be the primary reason for the marked differences in CpG-ODN 1826-induced activation of the two TLR9s.

**Table 5:** TLR9-ODN CpG interfaces

**Protein**	**Nucleic Acid**	**Interface area (Å**^**2**^**)**	**Interface residues**		**H-bonds**	**Hydrophobic contacts**
			***N*aa**	***N*nuc**		
mTLR9	ODN CpG	1945	31	13	7	53
hTLR9	ODN CpG	1790	25	12	7	29

Similarly to hTLR9, residues within LRR11 in mTLR9 provided more direct contacts to the CpG ODN molecule. In contrast to hTLR9, Arg-335 within LRR11 in mTLR9 increased the hydrophobic interactions between mTLR9 and CpG ODN 1826 (Table 3). This residue was likely one of the main reasons why mTLR9 was more responsive to ODN 1826.

## Discussion

Although TLR9 is known to be the receptor for CpG-DNA by directly engaging the ligand, the precise sequence and structural requirements of TLR9 to promote binding are unknown. Moreover, it is unclear which residues recognise and bind the CpG DNA ligand and which LRR is the binding site(s) for CpG DNA or is involved in the binding site(s). There are no crystal structure data for TLR9; in order to investigate how TLR9 interacts with CpG ODN, the 3D structure of the TLR9 ECD was constructed using homology modelling. Homology modelling, also referred to as comparative modelling, is currently the most accurate computational method for protein structure prediction. A limitation of homology modelling is that the quality of the predicted model strongly depends on the sequence identity between the target and the template.

The predicted hTLR9 ECD-CpG ODN model suggested that hydrogen bond interactions and hydrophobic contacts between hTLR9 and ODN 1826 play key roles in their recognition and binding. Residues within LRR11 in hTLR9 provided more interactions with CpG ODN 1826 than other LRRs. This result indicates that LRR11 may be the main region of hTLR9 binding to the CpG ODN. This conclusion is in agreement with results from our previously published experimental data [17].

The model not only identified the binding sites for CpG-ODN but also provided a structural basis for the species-specific ligand binding. In our observations, the mTLR9 ECD-CpG ODN complex had greater interface area compared with the hTLR9 ECD-CpG ODN complex and provided more hydrophobic interactions. This result could explain why CpG ODN 1826 activates mTLR9 more potently than hTLR9.

In this study, a modelling approach was successfully applied to generate the structures of hTLR9 and mTLR9 ECDs. The predicted model of the TLR9-ODN complex provides us with features that are consistent with the experimental data and can be considered a rough approximation of how TLR9 might interact with its nucleic acid substrate. The docked TLR9-ODN complex model provides a structural framework for interpreting experimental data and further understanding the TLR9 signal transduction process. Finally, these results open new avenues for the computer-aided design of potential inhibitors or antagonists of the CpG DNA–TLR9 signalling pathway.

## Conclusion

By applying molecular modelling and protein docking methods, three-dimensional structures of two TLR9-CpG ODN complexes were built. With these structures, we obtained useful information about the detailed interaction between TLR9 and the ODN ligand. These models provide a novel picture of CpG DNA-TLR9 recognition and binding and elucidate the mechanism underlying the TLR signal transduction process.

## Methods

### Data sets

Sequences of human TLR9 and mouse TLR9 in FASTA format were retrieved from the public database UniProt/ExPASy (Swiss Bioinformatics Resource) http://expasy.org/tools/[26], having accession numbers of Q9NR96 and Q9NR96 respectively.

CpG ODN 1826 (CpG ODN, 5^′^-TCCATGACGTTCCTGACGTT-3^′^, the optimal immunostimulatory murine sequence) was used in this study.

### Molecular modelling

Searches for reference proteins, sequence alignments and homology modelling were performed in Discovery Studio 2.5 (Accelrys Inc.). A BLAST search of the PDB indicated that the crystal structures of human TLR3, TLR2 and TLR4 ECD were suitable template models for the TLR9 ECD. Although the full-length sequence identity between the TLR9 and the templates is relatively low (26–28%), the presence of highly conserved LRR motifs in the target and templates suggests that the comparative homology modelling of the TLR9 ECD using the TLR3, TLR2 and TLR4 ECD structure was appropriate [27]. For better accuracy, the three-dimensional structures of the ectodomains of TLR9 were created by I-TASSER, which combines the methods of threading, ab initio modelling, comparative modelling and structural refinement. The I-TASSER server is an online platform for protein structure and function predictions. The I-TASSER server employs a fragment-based method. Here, a hierarchical approach to protein structure modelling is used in which fragments are excised from multiple template structures and reassembled based on threading alignments [28]. 3D models are built based on multiple-threading alignments by LOMETS and iterative TASSER assembly simulations. I-TASSER (as ‘Zhang-Server’) was ranked the No. 1 server for protein structure prediction in recent CASP7, CASP8 and CASP9 experiments [29,30].

The final templates selected by I-TASSER were the TLR3 crystal structure (PDB code 2a0z), TLR4 crystal structure (PDB code 3fxi), TLR3 crystal structure (PDB code 1ziw), TLR4 crystal structure (PDB code 2z64), and TLR2 crystal structure (PDB code 2Z7X).

The quality of the predicted structure was evaluated using the Verify Protein (Profiles-3D) protocol. Profiles-3D Verify checks the validity of a protein structure (e.g., a homology model) by measuring the compatibility score of each residue in the given 3D environment. Scores for each residue and the whole protein are reported, and the expected high and low scores for a protein of the same size are given as a reference point [31].

We employed the Biopolymer tools in Discovery Studio 2.5 to build the ODN model. Because TLR9 binds only to single-stranded molecules [32] and the immunostimulatory activity of CpG-DNA depends on its single-stranded (ss) character [33], we modelled the ODN structure as a single-stranded DNA molecule. The single-stranded ODN molecule was generated in B-form using standard helix parameters.

### Docking

The program Hex version 6.12 [34] was applied to perform a docking search. In Hex’s docking calculations, the TLR9 ECD was defined as the receptor, and ODN was used as the ligand. The shape plus electrostatic correlation algorithms were used. Other parameters used for the docking process were set to the default values.

The parameters that were used for the docking process via HEX docking software were as follows:

• Correlation type – Shape plus electrostatic correlation

• FFT Mode – 3D fast life

• Grid Dimension – 0.6

• Receptor range – 180

• Ligand Range – 180

• Twist range – 360

• Distance Range – 40

### Protein–DNA interaction analysis

The interface area of a complex is derived from its atomic coordinates by computing its accessible surface area in solvent and subtracting it from the sum of the accessible surface areas of the isolated components [12]. Accessible surface areas were evaluated with the program SURVOL [35], which implements the Lee and Richards algorithm [36]. Group radii were from ref [37], and the radius of the water probe was 1.4 Å.

A hydrophobic contact is defined as a distance between carbon atoms shorter than 4.5 Å [38]. All hydrophobic contacts between nucleic acid residues and protein residues are listed.

Protein structure illustrations were generated with the PyMOL Molecular Graphics Software [39].

## Abbreviations

TLR: Toll-like receptor; NF-κB: Nuclear factor-κB; LRR: Leucine rich repeat; ECD: Ectodomain.

## Competing interests

The authors declare that they have no competing interests.

## Authors’ contributions

JY, HZ and LL formulated the study. WZ and YL performed the research. BL and YG analysed the data. XP and ZQ participated in the analysis and discussion. JY wrote the manuscript. All authors read and approved the final manuscript.
